# Evolution of pyrrolizidine alkaloid biosynthesis in Apocynaceae: revisiting the defence de‐escalation hypothesis

**DOI:** 10.1111/nph.15061

**Published:** 2018-02-26

**Authors:** Tatyana Livshultz, Elisabeth Kaltenegger, Shannon C. K. Straub, Kevin Weitemier, Elliot Hirsch, Khrystyna Koval, Lumi Mema, Aaron Liston

**Affiliations:** ^1^ Department of Biodiversity, Earth, and Environmental Sciences Academy of Natural Sciences of Drexel University 1900 Benjamin Franklin Parkway Philadelphia PA 19103 USA; ^2^ Biochemical Ecology and Molecular Evolution Botanical Institute Christian‐Albrechts University Kiel Olshausenstrasse 40 24098 Kiel Germany; ^3^ Department of Biology Hobart and William Smith Colleges Geneva NY 14456 USA; ^4^ Department of Botany & Plant Pathology Oregon State University 2082 Cordley Hall Corvallis OR 97331 USA

**Keywords:** alkaloids, biosynthetic pathway, coevolution, gene duplication, plant–herbivore interactions, secondary metabolism

## Abstract

- Plants produce specialized metabolites for their defence. However, specialist herbivores adapt to these compounds and use them for their own benefit. Plants attacked predominantly by specialists may be under selection to reduce or eliminate production of co‐opted chemicals: the defence de‐escalation hypothesis.
- We studied the evolution of pyrrolizidine alkaloids (PAs) in Apocynaceae, larval host plants for PA‐adapted butterflies (Danainae, milkweed and clearwing butterflies), to test if the evolutionary pattern is consistent with de‐escalation.
- We used the first PA biosynthesis specific enzyme (homospermidine synthase, HSS) as tool for reconstructing PA evolution. We found *hss* orthologues in diverse Apocynaceae species, not all of them known to produce PAs. The phylogenetic analysis showed a monophyletic origin of the putative *hss* sequences early in the evolution of one Apocynaceae lineage (the APSA clade). We found an *hss* pseudogene in *Asclepias syriaca*, a species known to produce cardiac glycosides but no PAs, and four losses of an HSS amino acid motif. APSA clade species are significantly more likely to be Danainae larval host plants than expected if all Apocynaceae species were equally likely to be exploited.
- Our findings are consistent with PA de‐escalation as an adaptive response to specialist attack.

## Introduction

Flowering plants and their insect herbivores are a major focus of research into evolutionary links between ecological interactions and species and phenotypic diversity (Fraenkel, 1959; Ehrlich & Raven, 1964; Futuyma & Agrawal, 2009). They are remarkable for: their species richness – together, the two lineages comprise about half of described macroscopic species; the diversity of known plant secondary metabolites with > 100 000 distinct molecular structures, many of which are implicated in defence; the high degree of host‐specificity among herbivorous insects; and the (relative) phylogenetic conservatism of these traits in the interacting partners (Schoonhoven *et al*., 2005). The ‘escape and radiate’ model of coevolution (Ehrlich & Raven, 1964; Thompson, 1999) explains these observations by proposing an evolutionary sequence of chemical defence innovation permitting escape from herbivory and plant radiation, followed by evolution of a counter‐response, colonization and radiation in a clade of herbivores. Through multiple cycles of this ‘coevolutionary arms race’ (Berenbaum & Feeny, 1981), the model predicts evolutionary escalation in the diversity and potency of plant defences (Berenbaum, 1983; Vermeij, 1994; Becerra *et al*., 2009; Becerra, 2015). Recent research has highlighted gene duplication and neofunctionalization as a key mechanism in the evolution of novel secondary metabolites and escape from herbivory (Edger *et al*., 2015).

Evolutionary escalation models (Vermeij, 1994) do not account for adapted specialist herbivores’ use of their host plants’ secondary chemicals to increase their own fitness, as cues for host plant location, and/or by sequestering the chemicals for use in defence against their predators (Petschenka & Agrawal, 2015). Plants attacked predominantly by specialists may be under selection to reduce production of the co‐opted chemicals (to de‐escalate them) in favour of other defensive metabolites or strategies such as tolerance (ability to regrow after defoliation), low nutritional quality, mechanical defences and/or indirect defences via traits that increase predator fitness (van der Meijden, 1996; Joshi & Vrieling, 2005; Lankau, 2007; Ali & Agrawal, 2012; Cogni *et al*., 2012). Evidence of macroevolutionary de‐escalation of secondary metabolites is rare, but known (Agrawal *et al*., 2008, 2015; Becerra *et al*., 2009).

**Figure 1:**
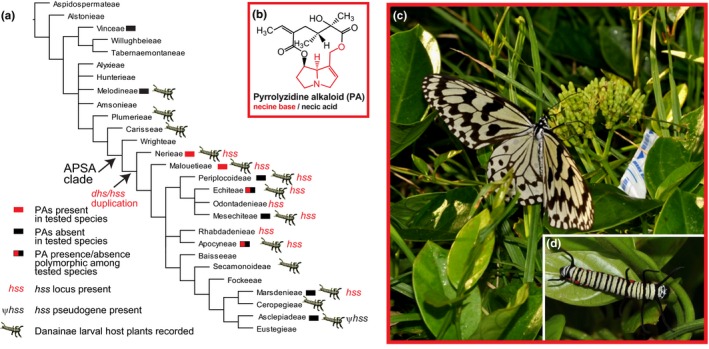
(a) The current best estimate phylogeny of 27 lineages of Apocynaceae (Livshultz *et al*., 2007; Simões *et al*., 2007; Straub *et al*., 2013, 2014) with the known distribution of pyrrolizidine alkaloid (PA) positive and negative species (Burzynski *et al*., 2015; Colegate *et al*., 2016) (Supporting Information Table S1). Tribes and subfamilies follow the classification of Endress *et al*. (2014). The occurrence of putative homospermidine synthase (*hss*) loci (Fig. 2b; Table S1) and the inferred location of the deoxyhypusine synthase (*dhs*)/*hss* duplication are indicated. Caterpillars indicate lineages with reported larval host plants for Danainae (Robinson *et al*., 2010) (Tables S5, S6). (b) Pyrrolizidine alkaloids (PAs) such as parsonsinine are known from species of four distinct lineages of Apocynaceae including (c, d). *Parsonsia alboflavescens* (Echiteae), the larval host plant of the danaine *Idea leuconoe* (c, d). (d) *Idea leuconoe* sequesters PAs from *P. alboflavescens* via larval feeding, hypothesized as the ancestral mode of acquisition (Honda *et al*., 1997), whereas most Danainae acquire PAs via (c) adult feeding on PA sources such as the nectar of *P. alboflavescens*. APSA clade, Apocynoideae Periplocoideae Secamonoideae Asclepiadoideae clade; ψ*hss*, homospermidine synthase pseudogene.

Here, we look for evidence of macroevolutionary de‐escalation in the dogbane family (Apocynaceae). Apocynaceae are best known for their production of monoterpenoid indole alkaloids, steroidal alkaloids and cardenolides (Endress *et al*., 1990; Agrawal *et al*., 2012). However, Apocynaceae also produce pyrrolizidine alkaloids (PAs) in species of four tribes of the APSA (Apocynoideae, Periploicoideae, Secamonoideae, Asclepiadoideae) clade: Echiteae, Apocyneae, Malouetieae and Nerieae (Burzynski *et al*., 2015; Colegate *et al*., 2016) (Fig. 1a). It is unknown if this scattered occurrence of PAs results from independent origins or from secondary loss of an ancestral compound. The PAs (Fig. 1b) are among the best‐studied secondary metabolites mediating ecological interactions and plant defence (Hartmann & Witte, 1995; Trigo, 2011). Structurally, PAs are alkaloids consisting of a necine base moiety esterified with a necic acid (Hartmann & Witte, 1995). Biosynthesis has been studied in five of 12 flowering plant families that produce PAs (Hartmann & Witte, 1995; Hartmann, 2009; Langel *et al*., 2011). In each case, the first step is catalysed by homospermidine synthase (HSS), which evolved at least six times independently in these five families by duplication and subfunctionalization of deoxyhypusine synthase (DHS), an essential eukaryotic enzyme that catalyses the activation of eIF5A (eukaryotic translation initiation factor 5A) (Nurhayati & Ober, 2005; Ober & Kaltenegger, 2009; Kaltenegger *et al*., 2013). The functional change from DHS to HSS involves a shift in substrate preference. Whereas DHS transfers the aminobutyl moiety from spermidine to the eIF5A, HSS uses putrescine as aminobutyl acceptor. In *Ipomoea neei*, three functional amino acid substitutions were shown to convert DHS into a more HSS‐like enzyme by drastically reducing its activity with eIF5A (Kaltenegger *et al*., 2013).

Pyrrolizidine alkaloids are highly toxic, but multiple insect lineages have evolved mechanisms of PA tolerance and sequestration, and use PAs for their own defence against predators (Hartmann & Witte, 1995). The Lepidoptera subfamily Danainae *sensu lato* (milkweed and clearwing butterflies; Wahlberg *et al*., 2009; Brower *et al*., 2014) uses PAs for both defence and in mating. Danaine males synthesize mating pheromones from PAs (Boppré, 1990). *Danaus gilippus* uses them in courtship as honest advertisements of nuptial gifts of protective PAs that males transfer to females upon mating; females then transfer these PAs to the eggs (Dussourd *et al*., 1989). These traits may be shared by all danaines (Brower *et al*., 2010, 2014). The best‐known species of Danainae, the monarch, *Danaus plexippus*, is the only known species that does not produce PA‐derived pheromones; however, it does sequester PAs (Kelley *et al*., 1987). Despite this intensive use of PAs, most danaine species are reported to have larval host plants that lack PAs; instead the adults acquire PAs through pharmacophagy (Fig. 1d) (Boppré, 1984), that is, feeding primarily to obtain secondary chemicals rather than nutrients. Only a few species sequester PAs via larval feeding that are retained through metamorphosis: *Idea leuconoe* (Fig. 1c,d), proposed as a model of plesiomorphic Danainae (Honda *et al*., 1997), *Tellervo zoilus* (Orr *et al*., 1996) and *Tithorea harmonia* (Trigo & Motta, 1990). However, larvae of danaine species that have PA‐free host plants (e.g. *D. plexippus*,* D. gilippus*,* Methona themisto*,* Mechanitis polymnia*) can also sequester PAs which are experimentally applied to their host plants and transmit them to imagos (Trigo & Motta, 1990). Trigo & Motta (1990) interpreted this as evidence of ancestral larval feeding on PA‐containing host plants. By contrast, Boppré (1978) proposed that adult pharmacophagy and PA‐derived pheromones evolved first in an ancestral species that fed on PA‐free host plants and that the shift to PA‐containing larval host plants is derived.

### Hypothesized coevolution between Danainae and Apocynaceae

Edgar (1984) proposed that loss of PAs in many Apocynaceae species is an adaptation to the PA‐philic Danainae. He hypothesized that PA‐derived mating pheromones evolved when the danaines’ common ancestor fed on PA‐containing Apocynaceae host plants. Selection by these (and potentially other) PA‐adapted herbivores caused highly attacked host plant species to lose PAs, which in turn led to the evolution of PA pharmacophagy. Loss of PAs did not permit these Apocynaceae species to shake off Danainae, but it may have reduced the absolute fitness of these herbivores (and increased the plants’ fitness). Meanwhile, Apocynaceae species that suffered greater herbivory from PA‐susceptible herbivores than from Danainae, whether due to biogeography or ecology, continued to produce PAs, a scenario described as the ‘evolving community of herbivores’ hypothesis (Agrawal *et al*., 2008). Adult pharmacophagy is proposed as a coevolutionary response to PA loss in the host plants.

In the present study, we test predictions of the defence de‐escalation hypothesis of PA evolution in Apocynaceae. By identifying HSS, the first gene of the PA biosynthetic pathway in *Parsonsia alboflavescens*, a PA‐producing species and danaine larval host plant (Fig. 1c,d) and other Apocynaceae species, we aim to reconstruct the evolution of PA biosynthesis. We ask when did HSS (and PA biosynthesis) evolve in Apocynaceae; is there evidence of loss of HSS (and PA biosynthesis); and could loss of PAs in Apocynaceae have occurred under selection from Danainae?

## Materials and Methods

### Sampling, vouchers, sequence deposition

**Figure 2:**
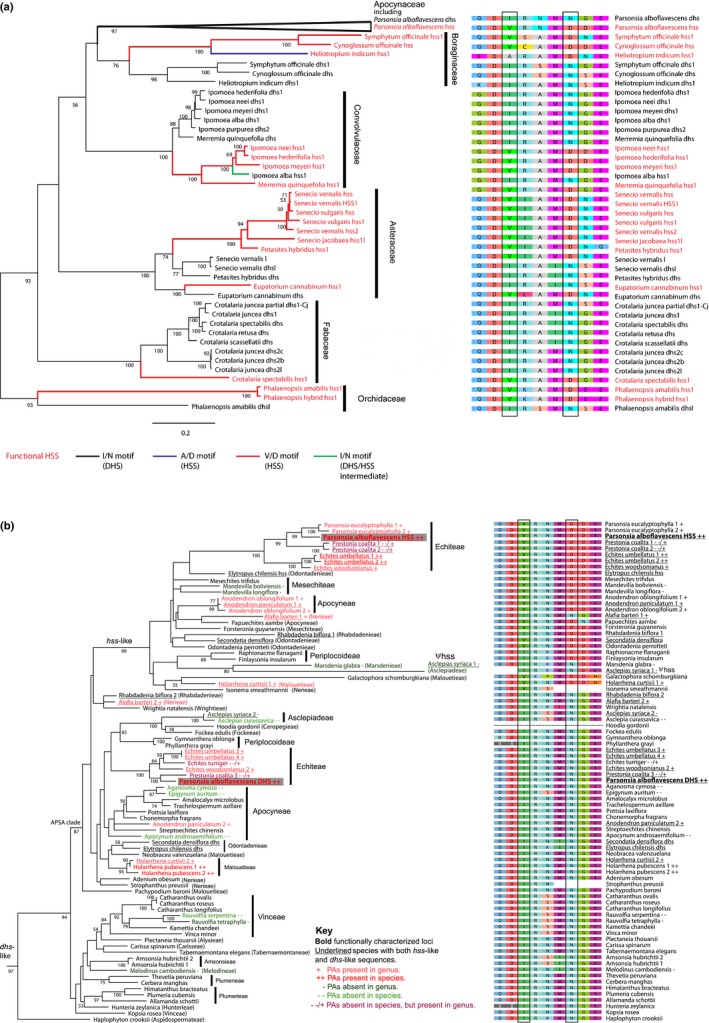
Maximum‐likelihood gene tree with bootstrap support of (a) all pyrrolizidine alkaloid (PA)‐producing genera with functionally characterized homospermidine synthase (HSS) loci and (b) candidate deoxyhypusine synthase (*dhs*)/*hss* loci from Apocynaceae. The evolution of the amino acid motif at positions 305 and 308 (numbered based on our alignment Supporting Information Notes S1) with maximum marginal likelihood is shown and the alignment is illustrated. (a) Functionally characterized HSS and DHS loci from across angiosperms. (b) Apocynaceae *hss*/*dhs*‐like loci. Red text, names of functionally characterized HSS sequences; black lines, isoleucine/asparagine (I/N) motif; red lines, valine/aspartic acid (V/D) motif; purple lines, alanine/aspartic acid (A/D) motif; blue lines, isoleucine/aspartic acid (I/D) motif; green lines, secondarily derived isoleucine/asparagine (I/N) motif. Underlined names, Apocynaceae species with both putative *dhs* and putative *hss*; +, PAs present in genus, unknown in species; ++, PAs present in species; ‐, PAs absent in genus, unknown in species; ‐ ‐, PAs absent in species; ‐ ‐/+, PAs absent in species but present in genus.

We included all functionally characterized *homospermidine synthase* (*hss*) and *deoxyhypusine synthase* (*dhs*) loci sequences from pyrrolizidine alkaloid (PA)‐producing genera, a total of 41 sequences from 20 species in 10 genera of Orchidaceae, Boraginaceae, Convolvulaceae, Asteraceae and Fabaceae (labelled in Fig. 2a; see Supporting Information Table S1) (Reimann *et al*., 2004; Nurhayati & Ober, 2005; Nurhayati *et al*., 2009; Ober & Kaltenegger, 2009; Kaltenegger *et al*., 2013; Irmer *et al*., 2015). We include the *hss1* of *Ipomoea alba* L. which groups phylogenetically with the functionally characterized *hss* sequences of Convolvulaceae but has a DHS‐like function (Kaltenegger *et al*., 2013). Within Apocynaceae, we sampled 64 species from 54 genera, four of five subfamilies and 21 of 25 tribes. We sampled six of seven genera with species reported to produce PAs (*Echites* P. Browne, *Parsonsia* R. Br., *Prestonia* R. Br., *Anodendron* A. DC., *Holarrhena* R. Br. and *Alafia* Thouars) and eight of 18 genera that have been tested and reported not to produce PAs (Burzynski *et al*., 2015; Colegate *et al*., 2016). We analysed three species that have been reported to contain PAs (*Echites umbellatus* Jacq., *Parsonsia alboflavescens* (Dennst.) Mabb. and *Holarrhena pubescens* Wall. ex G. Don) and two species from PA‐positive genera reported to lack PAs, *Echites turriger* Woodson and *Prestonia coalita* (Vell.) Woodson (Burzynski *et al*., 2015). Furthermore, we included 40 genera whose PA status has not been tested. See Fig. 1(a) for the distribution of PAs among Apocynaceae tribes, Fig. 2(b) for status of each sampled species and genus, and Table S1 for vouchers, GenBank accession numbers and PA status.

### Identification of cDNAs for *hss* and *dhs* in *P. alboflavescens*

Plants were grown from seed in anexic culture at the Botanical Gardens, Kiel, and shoot and root tissues harvested and frozen at −80°C until processing. Methods for plant culture, RNA extraction, cDNA synthesis and sequencing followed published methods (Ober & Hartmann, 1999; Kaltenegger *et al*., 2013). cDNAs were synthesized with Superscript™ II Reverse Transcriptase (Invitrogen). To identify *hss* and *dhs* homologues, a touchdown PCR of cDNA template was accomplished with *Taq* polymerase and degenerate primers P3for and P4rev in 40 cycles with annealing temperature declining from 60 to 45°C by 0.5°C every cycle. The 3′ and 5′ ends of the cDNAs were amplified using RACE technique (Life Technologies, Carlsbad, CA, USA). The obtained sequences were used to design primers to amplify the complete ORFs for subsequent heterologous expression of the recombinant protein in *Escherichia coli* (details in Tables S2, S3).

### Heterologous expression and functional characterization of candidate HSS and DHS from *P. alboflavescens*

Putative DHS and HSS encoding sequences containing the ORFs were cloned into expression vectors (Novagen™ pET22b (Millipore Sigma, Billerica, MA, USA) with an artificial C‐terminal hexahistidine (6xHis) tag extension), transformed into *E. coli* BL21 (DE3), and the proteins were purified and quantified according to previously described methods (Ober & Hartmann, 1999; Kaltenegger *et al*., 2013). HSS and DHS function were characterized using radiolabelled putrescine and recombinant eIF5A precursor protein from *Senecio vernalis*, respectively. EIF5A from *S. vernalis* is aminobutylated efficiently by DHSs from multiple angiosperm species (Reimann *et al*., 2004). Enzyme activity assays were conducted as described by Kaltenegger *et al*. (2013) following the methods of Ober & Hartmann (1999).

### Sequence database queries

The GenBank Nucleotide database (NCBI Resource Coordinators, 2017) and three transcriptome databases: 1KP (Johnson *et al*., 2012; Matasci *et al*., 2014; Wickett *et al*., 2014; Xie *et al*., 2014), Medicinal Plant Genomics Resource (Góngora‐Castillo *et al*., 2012a,b) and Phytometasyn (Xiao *et al*., 2013), were queried with keyword or BlastN searches to find candidate *dhs* and *hss* from PA‐positive angiosperm genera and from Apocynaceae. The *hss* and *dhs* orthologues in the *Asclepias syriaca* genomic database and transcriptome assemblies (Weitemier *et al*., 2018) were identified using Blat (Kent, 2002) (details in Table S4). When multiple identical or near identical sequence variants were obtained for a species, as identified by zero or near‐zero length branches in preliminary analyses, and they formed strongly supported clades (BS 99–100%) (data not shown), one to two sequences with the longest intact open reading frames (ORFs) were retained per species.

### Primer design

The alignment of sequences from GenBank, 1KP, the Medicinal Plant Genomics Resource and cDNAs from *P. alboflavescens* was used to design degenerate primers spanning exons 2 to 6. Additional primers against exon 1 were designed to confirm the presence of a deletion in the *hss* assembly from the *A. syriaca* genomic database using the alignment of sequences downloaded from all four databases and cDNAs from *P. alboflavescens*. All primers are in Table S3.

### DNA extraction, PCR, cloning and Sanger sequencing

Methods for DNA extraction are described in Livshultz *et al*. (2007). We used a nested PCR approach, first amplifying the largest possible segment spanning exons 2–6 using primers ‘degApo1for’ and ‘degApo1rev’ or exons 1–6 using ‘DHSHSS_ex1_Fa’ and ‘degApo1rev’ (Table S3), and then using the resulting PCR product (unpurified) as the template for PCR reactions with various combinations of internal primers, most often ‘degApo2for’ and ‘degApo2rev’ spanning exons 2–6. PCR was conducted in 15‐μl reactions consisting of 7.5 μl of Apex™ Taq DNA Polymerase Master Mix with 1.5 μl MgCl_2_ (Bioresearch Products, North Liberty, IA, USA), 4.25 μl of water, 0.4 μl of 200 mg ml^−1^ BSA and 1.5 μl each of the forward and reverse primers (10 μM stocks), with 0.5–1 μl of unquantified DNA extract or first step PCR reaction as template. Negative controls were always included. Reactions were performed in an Eppendorf MasterCycler ep Gradient 5341 thermal cycler for 3 min at 94°C, followed by 35 cycles of 1 min at 94°C, 1 min at 55°C, and 3.5 min (1^st^ step) or 3 min (2^nd^ step) extension at 72°C, ending with 10 min at 72°C. PCR products were visualized via agarose gel electrophoresis. Gel bands were purified using the Zymoclean™ Gel DNA Recovery Kit (Zymo Research, Irvine, CA, USA) according to manufacturer's instructions. PCR products were cloned with the pGEM‐T kit (Promega), and transformed into XL1‐Blue competent cells (Stratagene, La Jolla, CA, USA). White colonies were PCR‐screened with plasmid primers M13F and M13R and the resulting PCR products were Sanger‐sequenced with plasmid and internal primers. Sanger sequencing was conducted by Functional Biosciences (Madison, WI, USA).

### Sequence assembly

Contigs from the Sanger‐sequenced products were assembled using Wisconsin Package of Genetics Computer Group (GCG v.11.1) or Geneious v.5.6. *Asclepias syriaca* contigs containing *hss* and *dhs* orthologues were manually screened for possible misassembly by mapping sequence reads from the original genome assembly (Weitemier *et al*., 2018) onto assembled contigs using Bbmap v.35.85 (Bushnell, 2016) (‘slow’ mode, minimum identity = 99%) and screening for breaks in coverage.

### Exon annotation and alignment

Exon annotation and alignment was done with Geneious v.5.6. Exon boundaries were identified using the *hss* cDNA and gene from *P. alboflavescens* as a reference. Sequences from the *A. syriaca* genome were annotated manually in Geneious v.9.1.5, informed by transcript evidence, *ab initio* gene predictions (described in Weitemier *et al*., 2018), canonical splice site motifs, and similarity to *dhs* sequence from *A. curassavica*. To identify the 3′ end of the 7^th^ exon, the introns were spliced out and the 3′ end of the ORF adjusted to maximize the length of the transcript before the stop codon. The global alignment was made by splicing out the introns, translating the exons in frame with the consensus, and aligning exonic sequences using the Geneious translation align algorithm in Geneious v.10.2.3 using the Blosum62 cost matrix, a gap opening cost of 12, a gap extension cost of 3, and two rounds of refinement (Notes S1).

### Identification of potential pseudogenes and sequencing and assembly errors

Apparent nonsense or mis‐sense mutations or truncations of the candidate mRNAs were considered to be evidence of either pseudogenization or error. Potentially misassembled transcripts from online databases were identified via preliminary phylogenetic analyses. Conspecific sequences that formed strongly supported clades (BS = 99–100%) and were highly similar to assemblies with full intact ORFs were considered to be assembly errors and excluded. Electropherograms obtained via Sanger sequencing of genomic DNA were examined for poor sequence quality. Stop codons resulting from ambiguous sequence near the start or end of a sequence were eliminated by trimming. Truncated or missing exons resulting from incompletely sequenced regions were replaced by ‘Ns’ to restore the reading frame. A deletion in *A. syriaca hss* exon 2 was verified by PCR, cloning and Sanger sequencing from DNA extracted from a second accession (voucher: *Livshultz TL03‐33* deposited at BH).

### Maximum‐likelihood tree searches, model selection and bootstrap analyses

Maximum‐likelihood (ML) tree searches, model selection and bootstrap analyses were conducted with RAxML‐hpc v.8 (Stamatakis, 2006) on Xsede accessed via the CIPRES Science Gateway (Miller *et al*., 2009) accessed on 20 August 2017. The GTR plus GAMMA model of nucleotide substitution was applied in all steps of the analysis. The best partition model was selected by comparing the likelihood scores of analyses run under one and three (first, second and third codon positions) partition models with the Akaike information criterion. Branch lengths were always linked among partitions. Thorough ML tree searches were combined with 1000 rapid bootstrap replicates, command (‐f a).

### Ancestral state reconstruction and functional prediction

Ancestral DNA sequences were reconstructed with FastML (Ashkenazy *et al*., 2012) accessed via the server (fastml.tau.ac.il) on 20 August 2017, using the ML tree and marginal reconstruction to infer most likely sequences at internal nodes under the GTR plus GAMMA model and subsequently translated to amino acid sequences. The function of the reconstructed ancestral HSS enzymes was predicted based on the presence of two functionally characterized amino acid substitutions of HSS identified via site‐specific mutagenesis of the DHS sequence in *Ipomoea neei* (Kaltenegger *et al*., 2013). The value of these two substitutions for predicting protein function was validated via ancestral state reconstructions of functionally characterized HSS and DHS sequences across angiosperms.

### Host plant analysis

The HOSTS database (Robinson *et al*., 2010) was searched on 4 June 2017 using the search terms ‘Nymphalidae’ and ‘Apocynaceae’ or ‘Asclepiadaceae,’ and all host plant records for species of Danainae (tribes Danaini, Tellervini and Ithomiini) were downloaded (Table S5). We tabulated the number of plant species; records identified only to plant genus were included as distinct entries given the high species diversity of many of the genera and hence the likelihood that a taxon identified only to genus is a distinct species. We calculated the number of host species in each major lineage of Apocynaceae (classified as a tribe or subfamily, Fig. 1a) (Table S6). We conducted a χ^2^ test of goodness‐of‐fit to compare the number of host species in the APSA clade vs the outgroup lineages, to test the hypothesis that all species of Apocynaceae are equally likely to be detected as danaine hosts. Our estimates of the number of species not detected as hosts are based on the estimate that there are a total of 4500 species of Apocynaceae, 800 outside the APSA clade, 3700 in the APSA clade and 1200 in the tribe Asclepiadeae. Finally, we repeated the χ^2^ test twice after correcting for potential ascertainment biases by excluding host plant records for *Danaus plexippus*, the danaine species distributed over most of North America and the one with the most host plant records in the database; and excluding species of tribe Asclepiadeae, the tribe with the largest number of reported host species. Asclepiadeae are the most species‐rich lineage in North America and most species are herbaceous, both traits that may make caterpillar discovery more probable than on woody tropical plants.

## Results

### Identification of functional HSS and DHS in PA‐producing *P. alboflavescens*

Two sequences homologous to DHS were amplified from cDNAs of roots and shoots of *P. alboflavescens*. Biochemical characterization of the heterologously expressed proteins shows that the copy amplified from root cDNA encodes a DHS enzyme with high activity with both eIF5A (288 pkat mg^−1^) and with putrescine (274 pkat mg^−1^) as aminobutyl acceptor. The sequence amplified from shoot cDNA showed no activity with eIF5A but almost 10‐fold activity with putrescine as aminobutyl acceptor (2265 pkat mg^−1^) and thus proved to encode a HSS.

### HSS evolution in PA‐producing angiosperms

The multiple sequence alignment had 123 sequences and 1264 aligned base pairs (Notes S1). We reconstructed the gene tree of *hss* and *dhs* evolution under a three partition model which was preferred under the AIC criterion. The resulting topology (Fig. 2a,b) confirmed the already postulated six independent origins of HSS in monocots, Boraginaceae, twice in Asteraceae, Convolvulaceae and Fabaceae. Furthermore, a seventh independent origin of HSS was identified within Apocynaceae (Fig. 2b). All Apocynaceae sequences form a well‐supported clade (BS 97%) (Fig. 2a). All sequences from species that belong to the APSA clade (Fig. 1a) form a moderately supported clade (BS 87%) (Fig. 2b). Within the clade of APSA sequences, there is a well‐supported clade (BS 99%) which includes the functionally characterized *P. alboflavescens* HSS. Based on this topology, we classified all orthologues in this clade as putative HSS encoding sequences.

### Distribution of *dhs/hss* orthologues among Apocynaceae species

A total of 82 putative *dhs*/*hss* sequences were sampled from 64 Apocynaceae species, 13 from transcriptome databases, two from the *A. syriaca* genome, and 67 generated for this study (Table S1). Putative *hss* sequences were obtained only from genera in the APSA clade (Fig. 2b; Table S1). In 11 of 44 APSA clade species we identified both *hss* and *dhs*‐like sequences (underlined in Fig. 2b; Table S1); in 13 species we found *hss* only and in 20 species *dhs* only (Fig. 2b; Table S1). Only putative *dhs* sequences were obtained from the 20 non‐APSA Apocynaceae species (Fig. 2b; Table S1).

### Occurrence of putative *hss* genes and PAs in Apocynaceae

Species from all six sampled PA‐positive genera (*Echites*,* Parsonsia*,* Prestonia*,* Anodendron*,* Holarrhena* and *Alafia*) (labelled ‘+’ in Fig. 2b, see also Table S1) have a putative *hss* sequence. Two of the three PA‐positive species (labelled ‘++’ in Fig. 2b), *E. umbellatus* and *P. alboflavescens*, have both sequence types. In the third, *H. pubescens*, we identified only a DHS‐encoding sequence in the leaf transcriptome, but the closely related *H. curtisii* had both sequence types (Fig. 2b). Of the two species from PA‐positive genera that lack PAs, *E. turriger* had a *dhs*‐like sequence only whereas *P. coalita* had an *hss*‐like sequence only (labelled ‘‐ ‐/+’ in Fig. 2b, see also Table S1). Of species sampled from eight genera that have been tested and reported as lacking PAs (labelled ‘‐’ in Fig. 2b), six have only a putative *dhs* (*Rauvolfia serpentina* (Vinceae), *Melodinus cambodiensis* (Melodineae), *Epigynum auritum*,* Apocynum androsaemifolium* and *Aganosma cymosa* (Apocyneae), and *Asclepias curassavica* (Asclepiadeae)), three only a putative *hss* (*Mandevilla boliviensis* and *Mandevilla longiflora* (Mesechiteae), *Marsdenia glabra* (Marsdenieae)), and one had both (*A. syriaca* (Asclepiadeae)). We also detected a putative *hss* in nine species representing nine genera that had never been tested for PAs (*Papuechites aambe* (Apocyneae), *Galactophora schomburgkiana* (Malouetieae), *Forsteronia guyanensis* and *Mesechites trifidus* (Mesechiteae), *Isonema smeathmannii* (Nerieae), *Elytropus chilensis*,* Odontadenia perrotteti* and *Secondatia densiflora* (Odontadenieae), *Rhabdadenia biflora* (Rhabdadenieae), *Finlaysonia insularum* and *Raphionacme flanaganii* (Periplocoideae)) (Fig. 2b; Table S1).

### Identification of pseudogenes

Most apparent nonsense or mis‐sense mutations or truncations of the candidate sequences obtained from transcriptome data and Sanger sequencing turned out to be assembly or sequencing errors. These sequences were excluded from the analyses. However, the putative *Asclepias syriaca hss* has a 13‐bp deletion in exon 2 resulting in a stop codon 2 bp downstream and a dramatically truncated ORF. No other nonsense mutations were detected in this sequence but other evidence of pseudogenization includes deletion of the first 27 bp of ‘exon 1’ (relative to *A. curassavica*); the 3′ end of ‘exon 5’ is highly divergent; ‘exon 7’ has a noncanonical splice site; and the sequence is at the end of a very long branch (Fig. 2b). Furthermore, HSS was not detected in the *A. syriaca* shoot and bud transcriptomes.

### Repeated evolution of functionally characterized V/D amino acid motif

Ancestral state reconstruction indicates that two of the three amino acid substitutions which drastically reduced DHS activity in the mutagenized DHS of *I. neei*, evolved repeatedly across angiosperms (Fig. 2a,b). All 23 functionally characterized DHS sequences (including the *P. alboflavescens* DHS) have an isoleucine (I) at alignment position 305 and asparagine (N) at position 308 (numbering follows our alignment; Notes S1), whereas 18 of 19 unequivocally identified HSS encoding sequences have a valine (V) at alignment position 305 and an aspartic acid (D) at position 308 (Fig. 2a). The one exception is the HSS of PA‐producing *Heliotropium indicum* (Boraginaceae), which has an alanine (A) at 305. The characteristic V/D motif also is most likely to have been present in the ancestral sequence of all *hss*‐orthologues in Apocynaceae (Fig. 2b).

### Loss of V/D HSS motif within the *hss* clade of Apocynaceae and the *hss1* of *Ipomoea alba* (Convolvulaceae)

Based on the current best estimate of the phylogeny of the APSA clade (Fig. 1a), the V/D motif most likely was lost at least four times in the Apocynaceae *hss* clade: in the ancestral *hss* of *A. syriaca* and *M. glabra* (V/D to I/N), in the ancestral *hss* of subfamily Periplocoideae (*Raphionacme* and *Finlaysonia*) (V/D to I/D), in *A. barteri* (V/D to I/D), and in *R. biflora* (V/D to I/D) (Fig. 2b). The *hss1* gene of *I. alba* (Convolvulaceae), which functions more like a DHS, also has the *dhs*‐like I/N motif (Fig. 2a).

### Host plant analysis

We downloaded a total of 740 host plant records for species of Nymphalidae feeding on Apocynaceae. Of these, 726 records are from Danainae (a total of 67 butterfly species) exploiting 238 taxa representing 71 genera (Tables S5, S6). 702 records are for APSA clade genera and species, 14 records are from nine plant species that belong to three of the 11 outgroup tribes: Carisseae (one record), Plumerieae (10 records) and Melodineae (three records) (Fig. 1a), and 10 are not identified beyond family. The most frequently documented butterfly species is *D. plexippus* (119 of 726 records), and Asclepiadeae are most frequently documented as host plants (144 of 247 taxa). Ten of 16 primary APSA clade lineages (Fig. 1a) are represented among the host plant species, the exceptions are Wrightieae, Baisseeae, Odontadenieae, Rhabdadenieae, Eustegieae and Fockeeae. The χ^2^ test of goodness‐of‐fit indicates that the APSA clade (238 taxa) is significantly over‐represented among danaine host plants compared to other lineages (nine taxa), χ^2^ (2, *n *=* *4500) = 35.7, *P *<* *1 × 10^−5^. Neither exclusion of host records for *Danaus plexippus* (210 APSA, nine non‐APSA host taxa), χ^2^ (2, *n *=* *4500)* *=* *29.4, *P *<* *1 × 10^−5^, nor exclusion of records from tribe Asclepiadeae (94 APSA, 9 non‐APSA host taxa), χ^2^ (2, *n *=* *3300)* *=* *13.9, *P *=* *1.91 × 10^−4^, changed these results.

## Discussion

We tested predictions of a coevolutionary hypothesis (Edgar, 1984) of pyrrolizidine alkaloid (PA) evolution in Apocynaceae under selective pressure from herbivory from the PA‐adapted Danainae by reconstructing the evolution of the first locus of the PA biosynthetic pathway, *homospermidine synthase* (*hss*), and the distribution of danaine larval host plants in the family. Edgar (1984) proposed a sequence of three reciprocal adaptations: PA‐phily of Danainae as an adaptation to larval feeding on PA‐producing Apocynaceae host plants; loss of PAs as an adaptation of Apocynaceae to herbivory by PA‐philic Danainae; and PA‐pharmacophagy as a danaine adaption to loss of PAs in the Apocynaceae host plants. Tests of adaptive macroevolutionary hypotheses typically perform two kinds of analyses: a phylogenetic reconstruction to test if the proposed adaptive trait is evolutionarily derived; and a comparison of function to test if taxa with the proposed adaptation perform better than taxa that retain the ancestral state under the conditions that are hypothesized to have selected for the adaptation (Coddington, 1988; Baum & Larson, 1991; Martins, 2000). Here we focus on the evolutionary pattern of PA biosynthesis to address the second of Edgar's (1984) hypotheses.

### When did HSS evolve in the Apocynaceae?

We show a single origin of the *hss* locus in Apocynaceae early in the diversification of the APSA (Apocynoideae, Periploicoideae, Secamonoideae, Asclepiadoideae) clade (Figs 1a, 2b). Based on current sampling, this occurred after divergence of Wrightieae (represented by *Wrightia natalensis*), because no *hss* sequence was obtained from this tribe, whereas both a putative *hss* and PAs are present in *Alafia* (Nerieae, the next diverging tribe) (Figs 1a, 2b). Although gene discovery based on transcriptomes and PCR amplification may miss loci that are present, as evidenced by the fact that we failed to detect the essential *deoxyhypusine synthase* (*dhs*) gene in 13 of 64 species (Fig. 2b), the support for monophyly of all *dhs* and *hss* sequences from the APSA clade species (Fig. 2b) suggests that orthologues of the putative *hss* we discovered are not likely to be found outside this clade.

But is the *hss* locus per se evidence for PA biosynthesis? We show that two functionally characterized amino acid substitutions which might be involved in the change of substrate specificity and thus activity from DHS to HSS (Kaltenegger *et al*., 2013) are most likely present in the ancestral sequence of the APSA *hss* clade (Fig. 2b). Although further functional analyses are necessary, the repeated occurrence of these substitutions in most characterized HSS sequences (Fig. 2a) supports their functional importance in the evolution of HSS activity. Thus, not only the locus, but also the enzymatic properties of the first enzyme of PA biosynthesis, and with this an essential prerequisite for PA biosynthesis, most likely evolved early in the evolution of the APSA clade. Although these results agree with the prediction that the ancestor of most APSA clade species produced PAs, reconstructing the evolution of enzymes catalysing later steps in PA biosynthesis is necessary to further test it.

### Is there evidence of loss of HSS and PA biosynthesis?

Evidence for a single origin of HSS (Fig. 2b) in the ancestor of most APSA clade species, in contrast to the rare and spotty reports of PAs in only four of sixteen lineages (Fig. 1a), suggests multiple independent losses of PAs. The presence of an *hss* pseudogene in *Asclepias syriaca* and the independent loss of the HSS‐specific V/D motif in additional *hss* sequences (Fig. 2b), which might point to a change or even loss of HSS function, is consistent with this hypothesis. In experimental mutagenesis of *Ipomoea neei dhs*, the I to V mutation resulted in a slightly improved HSS activity, whereas the N to D mutation reduced DHS activity (Kaltenegger *et al*., 2013). Of note, the *hss1* gene of *I. alba*, a species which lacks PAs, has the I/N motif (Fig. 2a), and the encoded enzyme shows an intermediate substrate preference, it can readily catalyze the HSS and DHS reactions (Kaltenegger *et al*., 2013). Further functional studies are necessary to understand the effect of these substitutions in more detail.

Given the current understanding of phylogenetic relationships in the APSA clade (Fig. 1a), assuming that PAs are confined to the four currently known tribes, and an early origin of PAs, our results imply a minimum of five independent losses, in Periplocoideae, in Odontadenieae plus Mesechiteae, in Rhabdadenieae, in the clade that includes Baisseeae and Asclepiadeae, and in *Alafia barteri* (Fig. 1a). Although *Alafia* is one of the PA‐producing genera (Pais *et al*., 1971; Colegate *et al*., 2016), *A. barteri* has not been tested for PAs. *Alafia* may be a third genus, alongside *Echites* and *Prestonia,* where PA presence is apparently polymorphic among species (Burzynski *et al*., 2015). A study to test the correlation of *hss* genotype and PA phenotype among closely related species with and without PAs would illuminate the functional importance of the V/D motif and help identify other functionally important amino acid motifs.

We discovered *hss* genes with intact V/D motifs in species and genera which were reported to lack PAs (*Prestonia coalita* and *Mandevilla*) (Burzynski *et al*., 2015) (Figs 1a, 2b; Table S1). There are several possible causes for these apparent discrepancies and different causes may apply in each case: these *hss* loci may be pseudogenes; although we did not discover any nonsense mutations in the gene regions we sequenced, our sequences are missing all of exons 1 and 7, and most of exons 2 and 6; these *hss* loci may be silenced, either not transcribed or not translated; and although these *hss* loci possess the V/D motif characteristic of HSS enzymes, they still may have lost their HSS function via nonsynonymous substitutions at other functionally important sites. The V/D motif is likely to be necessary but not sufficient to confer HSS activity because in mutagenesis experiments with *dhs* of *I. neei*, it is insufficient to convert a DHS to an enzyme with full HSS activity (Kaltenegger *et al*., 2013). The loss of PA biosynthesis may be caused by pseudogenization of an unknown locus downstream in the PA biosynthetic pathway; or these species may indeed produce PAs but PA biosynthesis may be polymorphic among populations of a species or among species of a genus; or PA accumulation may be organ‐specific. In at least three species of Apocynaceae, PAs are much more concentrated in roots than in shoots, whereas most testing for PAs is conducted on aboveground organs (Burzynski *et al*., 2015; Colegate *et al*., 2016).

### Could loss of PAs in the APSA clade have occurred under selection from Danainae?

For Danainae to function as an agent of selection on APSA clade defences, they must affect host plant fitness, which is more likely if they interact frequently. We show that APSA clade species are significantly more likely to be reported as danaine larval host plants than species of earlier diverging lineages (Fig. 1a; Table S6) and that this does not change when we correct for potential ascertainment biases, indicative that the APSA clade is the Apocynaceae lineage that interacts most frequently with Danainae. Current age estimates for the two groups suggest that they could have been interacting for the entire history of the APSA clade. Phylogenetic dating analyses suggest that the Danainae crown clade, estimated to have begun to diversify 55 million yr ago (Ma) (51–77 Ma 95% confidence interval) (Wahlberg *et al*., 2009), is older than the stem lineage of the APSA clade, estimated divergence 48 Ma (47–51 Ma 95% confidence interval) (Ribeiro *et al*., 2014).

Even when we have a comprehensive picture of PA evolution across the APSA clade, it will not tell us that selection from Danainae is the cause of PA losses, rather than interactions with other PA‐philic herbivores that also exploit these plants, for example, species of Erebidae subfamily Arctiinae (Robinson *et al*., 2010; Zaspel *et al*., 2014) and Chrysomelidae (Hartmann *et al*., 2001). Indirect evidence may be obtained from comparing the distribution of larval host plant records among APSA lineages. If lineages that are more highly exploited by Danainae have lost PAs, whereas less exploited lineages that suffer more from other adapted herbivores and generalist have retained them, it would be consistent with Danainae as the most important agents of selection for PA loss. Population‐level studies of PA‐polymorphic species would be very useful to determine if the fitness cost of herbivory by different insect groups varies with PA status, as has been shown experimentally for other defensive secondary metabolites (Lankau, 2007).

### Why cardenolides and not PAs?

Study of host–herbivore interactions between Danainae and Apocynaceae mediated by secondary metabolites has been mostly focused on cardenolides, not PAs (Dobler *et al*., 2012; Agrawal *et al*., 2015; Petschenka & Agrawal, 2015). Of the genera exploited by Danainae, 20 have cardenolides, 22 lack them and 29 have not been tested (Agrawal *et al*., 2012) (Table S6). Why are Apocynaceae taxa that host danaine larvae more likely to have cardenolides than PAs based on current knowledge? Dose‐dependent fitness costs to adapted herbivores have been well‐documented for cardenolides, reviewed in Agrawal *et al*. (2012). By contrast, studies on the moth *Utetheisia ornatrix* (Lepidoptera: Erebidae: Arctiinae), an adapted PA‐sequestering herbivore of *Crotalaria* (Fabaceae), suggest no fitness costs from increasing concentrations of PAs in their food (Cogni *et al*., 2012) and a preference for higher concentrations under certain circumstances (Hoina *et al*., 2013). Likewise, experiments with specialist and generalist herbivores on *Cynoglossum officinale* (Boraginaceae) indicate that PAs are a defence only against unadapted generalists (Van dam *et al*., 1995). Ecological evidence is supportive, because populations of *Senecio jacobaea* (Asteraceae) introduced outside the range of their adapted herbivores increased PA production compared to populations within their native range (Joshi & Vrieling, 2005). We propose that APSA clade taxa attacked primarily by Danainae (and other adapted specialists) use cardenolides but not PAs, whereas species attacked primarily by generalists are more likely to retain PAs. Experimental evidence of absence of fitness costs from PAs to danaine larvae, and ecological evidence of the prevalence of generalist and specialist herbivores on Apocynaceae species that have retained vs those that have lost PAs are necessary to test this hypothesis.

### Conclusions

Evidence from evolutionary pattern is consistent with the predictions of the adaptive hypothesis of PA defence de‐escalation in Apocynaceae (Edgar, 1984). The *hss* locus, which catalyses the first step of PA biosynthesis, evolved once in Apocynaceae, early during the diversification of the APSA clade (Fig. 2b). *Hss* was pseudogenized in *A. syriaca*, a highly exploited danaine host plant, and an HSS amino acid motif has been lost multiple times (Fig. 2b), consistent with multiple independent losses of PAs. The APSA clade includes 98% of all known danaine host plants, and phylogenetic dating studies of Danainae and the APSA clade indicate that they may be of similar age, both consistent with the hypothesis that Danainae were an agent of selection for PA loss in this lineage. Better understanding of PA distribution, *hss* evolution, and functional characterization of additional *hss* genes in Apocynaceae will clarify how many times PAs have been lost and by what mechanisms. Studies of herbivores on closely related species that have retained PAs and those that have lost them will greatly illuminate how the ‘evolving community of herbivores’ (Agrawal *et al*., 2008) may have driven the evolution of secondary chemistry in this lineage. Reconstruction of the evolution of larval host plant chemistry in Danainae is necessary to test the other components of Edgar's (1984) overall coevolutionary hypothesis: that larval PA acquisition is ancestral and adult pharmacophagy derived.

## Author contributions

T.L. and E.K. planned and designed the research; T.L., E.K., E.H., K.K. and L.M. performed experiments and collected data; T.L., E.K., S.C.K.S. and K.W. analysed data; and T.L., E.K., S.C.K.S., K.W. and A.L. wrote the manuscript.

## Supporting information

Please note: Wiley Blackwell are not responsible for the content or functionality of any Supporting Information supplied by the authors. Any queries (other than missing material) should be directed to the *New Phytologist* Central Office.

**Table S1** Vouchers, Genbank accession and 1KP scaffold numbers, and PA status of sampled species
**Table S2** Amplification conditions for *hss* and *dhs* cDNAs from *Parsonsia alboflavescens*
**Table S3** Primers
**Table S4** Sequence database queries
**Table S5** Host plant records for Nymphalidae on Apocynaceae from the HOSTS database
**Table S6** Counts of Danainae host plant records per species of Apocynaceae in the HOSTS database, total number of host plant species recorded per tribe or subfamily, and cardenolide status of each host plant genus based on Agrawal *et al*. (2012)

**Notes S1** Alignment.
