# Supplementary material for: Evolution of pyrrolizidine alkaloid biosynthesis in Apocynaceae: revisiting the defence de‐escalation hypothesis
Source: New Phytol. 2018 Feb 26;218(2):762–73. doi: 10.1111/nph.15061 (PMC5873419; doi:10.1111/nph.15061)
Supplement: Supplementary file 3 [file NPH-218-762-s003.pdf]

***New Phytologist* Supporting Information**

Article title: Evolution of pyrrolizidine alkaloid biosynthesis in Apocynaceae: revisiting the defence de-escalation hypothesis
Authors: Tatyana Livshultz, Elisabeth Kaltenegger, Shannon C. K. Straub, Kevin Weitemier, Elliot Hirsch, Khrystyna Koval, Lumi Mema, Aaron Liston
Article acceptance date: 10 January 2018

The following Supporting Information is available for this article:

Table S1 Vouchers, Genbank accession and 1KP scaffold numbers, and PA status of sampled species (separate Excel file).

Table S2. Amplification conditions for *hss* and *dhs* cDNAs from *Parsonsia alboflavescens*.

Table S3. Primers

Table S4. Sequence database queries

Table S5. Host plant records for Nymphalidae on Apocynaceae from the HOSTS database (separate Excel file).

Table S6. Counts of Danainae host plant records per species of Apocynaceae in the HOSTS database, total number of host plant species recorded per tribe or subfamily, and cardenolide status of each host plant genus based on Agrawal *et al.* (2012) (separate Excel File).

Notes S1. Alignment (separate text file).

Table. S2 Amplification conditions for *hss* and *dhs* cDNAs from *Parsonsia alboflavescens*, including tissue source for cDNA of each locus, primers used for PCR and 3’ and 5’ RACE and PCR conditions. All primers are listed in Table S3.

**locus**	**source tissue**	**PCR primers**	**3' RACE primers**	**5' RACE primers**	**PCR conditions**
dhs	root	P3 for, P4 rev	P11	P23, P25	Taq DNA-Polymerase, touchdown PCR 60-45 °C, -0.5°C per Cycle, 40 cycles.
hss	shoot	P3 for, P4 rev	P12	P26, P28	Taq DNA-Polymerase, touchdown PCR 60-45 °C, -0.5°C per Cycle, 40 cycles.

Table S3 Primers, all written in the 5’ to 3’ orientation.

Primer name	primer sequence	primer location	primer direction	use
EK-degApo1for	TSYAVWGAWSARGAAARRGACCC	exon 2	forward	first step of nested PCR
EK-degApo2for	TTCACTTCAAATCTYRTTTCTTCHGGSGT	exon 2	forward	second step of nested PCR and sequencing
EK-degApo3for	AAAGGRYTRAATCGCATWGGTAAYTTRTTGG	Exon 3	forward	second step of nested PCR and sequencing
EK-degApo1rev	CCCCAGGABAYDGCTTCATCWGGDYG	exon 6	reverse	first step of nested PCR
EK-degApo2rev	CATCATRTTKGCATWRCAWAKRTGRTGCTT	exon 6	reverse	second step of nested PCR and sequencing
EK-degApo3rev	CCYCCRCCAAGAATWATYATTCCYGTCTTCC	Exon 6	reverse	second step of nested PCR and sequencing

DHSHSS_ex1_Fa	RAMAATHRARGGWTAYGATTTYRRHARWGGAG	exon 1	forward	first step of nested PCR
DHSHSS_ex1_Fb	TGGTYTCCACTGGHTTYCARGC	exon 1	forward	second step of nested PCR and sequencing
P3for	5’-GAR GAR GAY YTN ATH AAR TGY YT-3’		forward	PCR amplification from cDNA from *P. alboflavescens*
P4rev	5’-GAY GAR GCN GTN WSN TGG GG-3’		reverse	PCR amplification from cDNA from *P. alboflavescens*
P11for	5’-AAG ATG TTA GAA GAA CAA TTA TCT GAG AAT T-3’		forward	3' RACE dhs from *P. alboflavescens*
P12for	5’-TTC ATT CTT TCC GCA ATC CTG CAA GTT TAA TC-3’		forward	3' RACE hss from *P. alboflavescens*

P23	5’-GCA CAA TAT CAA TAA CTA AAC CA-3’		reverse	first step of semi nested 5' RACE for dhs of *P. alboflavescens*
P25	5’-TCC TTC CCA AGG CGT GAA ATC ACT TTA-3’		reverse	second step of semi nested 5' RACE for dhs of *P. alboflavescens*
P26	5’-TGA ACA ATA TCA ACG ATT AAA CTT-3’		reverse	first step of semi nested 5' RACE for hss of *P. alboflavescens*
P28	5’-CCT TCC CAA GGC GAG CGA TTA CTC T-3’		reverse	second step of semi nested 5' RACE for hss of *P. alboflavescens*
P36	5’-ATA TAC TGC AGA TGG GTG AAG CCA TGA AGC AAA ACC T-3’		forward	full length dhs from *P. alboflavescens*
P37	5’-ATA CTC GAG GTC TTT GGC GGA ATC ATC CCT CTT TC-3’		reverse	full length dhs from *P. alboflavescens*

P38	5’-ATA TAC TGC AGA TGG GTG ACG ACG CAA TCA GGC AGA-3’		forward	full length hss from *P. alboflavescens*
P39	5’-TAT ACT CGA GAT ATA TAT ACC CAA GAT ATT TTG TCC AGGT-3'		reverse	full length hss from *P. alboflavescens*
P40	5’-TAT AAT TAA TAT GGG TGA CGA CGC AAT CAG GCA GA-3’		forward	full length hss from *P. alboflavescens*
P41	5’-TAT AGA ATT CTT AAT ATA TAT ACC CAA GAT ATT TTG TCC AG-3'		reverse	full length hss from *P. alboflavescens*

Table S4 Sequence database queries. The website, query date and query parameters are given for queries that were used to obtain sequences from available databases.

**Source**	**website**	**dates queried**	**query**
Genbank Nucleotide Database	https://www.ncbi.nlm.nih.gov/genbank/	July 27, 2017	keyword search with "homospermidine synthase"
1KP	onekp.com	Dec. 18 & Dec. 20, 2013	BlastN with HF911522.1 and HF911523.1 (*Ipomoea alba dhs* and *hss*)
Medicinal Plant Genomics Resources	http://medicinalplantgenomics.msu.edu/	Dec. 23, 2013	BlastN with *Rauvolfia tetraphylla dhs* from 1KP database (scaffold-QEHE-2023261-*Rauvolfia_tetraphyla* [sic])
Phytometasyn	https://bioinformatics.tugraz.at/phytometasyn/	Oct. 31, 2014	BlastN with *Apocynum androsaemifolium dhs* from 1KP database (scaffold-YFQX-2069752-YFQX-Apocynum_androsaemifolium-2_samples_combined)
Asclepias syriaca genome	http://milkweedgenome.org/cgi-bin/gb2/gbrowse/Asclepias_syriaca/	May 25, 2016	nucleotide BLAT query with *dhs* (85% similarity) and *hss* (70% similarity) cDNAs from *Parsonsia alboflavescens*
